# Establishment of prediction model for mortality risk of pancreatic cancer: a retrospective study

**DOI:** 10.1186/s12911-024-02590-4

**Published:** 2024-06-27

**Authors:** Raoof Nopour

**Affiliations:** Department of Health Information Management, Student Research Committee, School of Health Management and Information Sciences Branch, Iran University of Medical Sciences, Tehran, Iran

**Keywords:** Pancreatic cancer, Mortality risk, Machine learning, Prediction model, Prognostic factors

## Abstract

**Background and aim:**

Pancreatic cancer possesses a high prevalence and mortality rate among other cancers. Despite the low survival rate of this cancer type, the early prediction of this disease has a crucial role in decreasing the mortality rate and improving the prognosis. So, this study.

**Materials and methods:**

In this retrospective study, we used 654 alive and dead PC cases to establish the prediction model for PC. The six chosen machine learning algorithms and prognostic factors were utilized to build the prediction models. The importance of the predictive factors was assessed using the relative importance of a high-performing algorithm.

**Results:**

The XG-Boost with AU-ROC of 0.933 (95% CI= [0.906–0.958]) and AU-ROC of 0.836 (95% CI= [0.789–0.865] in internal and external validation modes were considered as the best-performing model for predicting the mortality risk of PC. The factors, including tumor size, smoking, and chemotherapy, were considered the most influential for prediction.

**Conclusion:**

The XG-Boost gained more performance efficiency in predicting the mortality risk of PC patients, so this model can promote the clinical solutions that doctors can achieve in healthcare environments to decrease the mortality risk of these patients.

## Highlights

We developed machine learning models to predict the mortality risk of pancreatic cancer.

XG-Boost demonstrated more competency in predicting mortality risk.

Prognostic factors are essential for predicting the mortality risk of PC.

Based on the external validation results, the clinical applicability of the XG-Boost is almost efficient in other clinical environments.

Some lifestyle factors, such as smoking, have a significant role in predicting the mortality risk on this topic.

## Introduction

Pancreatic cancer (PC) refers to the uncontrollable growth of cells in the pancreas gland, causing a cancerous mass that can spread to other tissues in the body [1]. More than 90% of this cancer type is formed from ductal epithelium, namely pancreatic ductal adenocarcinoma [2]. Based on the GLOBOCAN reports (2020), it is estimated that PC has 466,003 cases, including 246,840 and 219,163 among men and women, respectively [3]. In addition to the highly invasive nature of PC, the lethality of this malignancy is high, especially in people aged 65 to 80 years [4]. This cancer type currently has the seventh rank of mortality pertained to cancer worldwide. It is also the fourth leading cause of mortality after colorectal, lung, and breast tumors in the USA and European nations [5]. Based on the American Cancer Society, the new cases related to PC and the mortality caused by this disease in the USA are 62,210 and 49,830, respectively [6].

Due to changing lifestyles, this malignancy has an upward trend in developing and developed countries. As a developing country, Iran ranks 11th among Asian countries in terms of the death rate caused by this disease [7]. This malignancy accounts for the 12th cause of cancer-related death in Iran [8]. It is projected that the mortality rate caused by colorectal cancer will be overtaken by PC by 2030, and PC will be the second leading cause of cancer deaths among other cancers [9, 10].

Despite advances in diagnostic tools and treatment, this malignancy still has a high rate of mortality, such that the five-year survival rate of PC is approximately 10.8%, indicating a worse prognosis of PC than other cancer types [11]. More specifically, despite some treatments, such as surgery or palliative types, being performed to decrease the mortality rate of PC, these methods haven’t been efficient in reducing the mortality caused by the disease due to performing at advanced stages [12]. On the other hand, the five-year survival rate of PC will reach 44% if this disease is diagnosed at earlier stages with localized tumors [13].

As more advanced therapy strategies have been established for reducing the mortality of PC, we require more predictive tools for oncological outcomes [14]. In other words, predicting the oncological outcomes of this disease based on the prognostic factors plays a crucial role in increasing PC survival by detecting the factors contributing to worsening the patients’ outcomes at earlier stages [15]. More specifically, early prediction of the mortality risk of PC based on these factors can significantly increase PC survival by modifying these factors at earlier stages [16, 17].

Previous studies have demonstrated the potential role of Machine Learning (ML) techniques in predicting some clinical aspects with high-performance efficiency [18–21]. They also revealed more predictive competency than some techniques, such as conventional statistical methods [22, 23]. Also, the predictive model using an ML approach has given us insight into the satisfactory performance efficiency associated with PC disease. Chen et al. constructed a predictive model for PC detection in the early stages using the XG-Boost algorithm. They applied 18,220 features from the Electronic Health Record (EHR), including clinical notes, procedures, prescriptions, and diagnostic data. The XG-Boost performance with an AU-ROC of 0.84 was satisfactory for prediction [24]. In another research, Chakraborty et al. used XG-Boost to predict PC patients. Based on their study results, The XG-Boost could predict the PC with an accuracy of 96.42%. Also, based on this algorithm, age, BMI, and smoking were recognized as top features for predicting PC [25]. Khan et al. established predictive models using the XG-Boost algorithm to predict PC in patients with new-onset diabetes in healthcare environments in the USA. The XG-boost revealed a performance of AU-ROC of 0.8 for separating the high-risk PC group among patients with new-onset diabetes [26].

Although deep learning (DL) techniques have favorable predictive performance when dealing with high-volume and unstructured data such as images, signals, or videos, ML techniques provide favorable predictive insights when dealing with structured clinical data [27, 28].

As mentioned above, early prediction of PC mortality risk based on prognostic factors significantly affects the survival rate of PC. In this study, we aim to establish a predictive model using prognostic factors to assess the mortality risk of PC by getting assistance from ML techniques. In this way, the high-risk mortality groups in PC patients would be detected at early stages, and various clinical solutions would be considered for these patients to increase survival based on these factors, especially the modifiable ones.

## Methods

This study, as a data-driven and retrospective approach, was conducted as follows:

### Study population

In this study, we utilized the 654 data of positive cases belonging to PC patients referred to Imam Khomeini Hospital in Tehran City from January 2019 to December 2023, which were stored in one electronic database with the Excel sheet format. In the current database, 201 and 453 cases were associated with the alive and dead cases, respectively, following the five years of PC diagnosis.

### Prognostic factors and outcome

The prognostic factors that existed in the database and were used for prediction purposes included age at diagnosis, gender, race, residence status, Body Mass Index (BMI), smoking, alcohol consumption, history of gastrointestinal cancer, history of other cancers, surgery, chemotherapy, radiotherapy, grade of tumor, tumor size, lymph node invasion, metastasis status, histological type, and vascular invasion. The outcome variable was the mortality status of PC patients, which was specified with the 0 and 1 codes associated with alive and dead cases, respectively.

### Database preparation

In this study, we first investigated and prepared the current database before establishing the predictive models for the mortality risk of PC based on ML techniques. In the first step, we confronted the redundancy in the cases, so any rows in the database associated with one patient were excluded from the study. In the second step, we investigated the lost data of cases in the current database. For the lost data, we had two situations. If the lost data existed in the outcome variable, the cases with this condition were excluded from further analysis. In the condition that the lost data were associated with the prognostic factors, we had two scenarios. If the cases had more than 10% lost data in their features, we removed those cases. In this respect, we selected this threshold to remove the cases due to keeping the bias minimal in ML techniques’ predictive performance. For the cases with less than 10% lost data, we filled them with the most similar records’ values obtained by the K-Nearest-Neighbor (KNN) algorithm.

### Feature selection

In the current study, we applied the feature selection technique to gain the most important prognostic factors for predicting the mortality risk of PC. In ML science, the feature selection technique selects the best features for the learning process. On the contrary, the irrelevant or redundant data would be eliminated from the dataset. It has some benefits in the ML process, including reducing the computational time for learning, preventing the algorithms’ overfitting during the learning process, enhancing the learning accuracy, and facilitating the better perception of data by ML algorithms [29]. To this end, we used binary logistic regression to score and choose the best features for predicting the mortality risk of PC. In this respect, the *P* < 0.05 was considered a threshold for selecting the features. This approach can be regarded as the wrapper approach of the feature selection technique. In this approach, data modeling occurs using an algorithm. Choosing the features based on this approach gives us a higher predictive capability than filter methods such as the Chi-square test based on the ranked features obtained [30, 31]. Furthermore, logistic regression selects features having a statistically significant hybrid correlation with the output class. The combination of logistic regression as a multi-variable selection strategy for feature selection and ML algorithms has a substantial role in enhancing the performance efficiency of these algorithms, and this subject has been shown in previous studies on biomedical research [32–34].

### Models development and validation

We developed the prediction models based on ML algorithms in Weka software V 3.9.1. In this respect, six chosen ensemble and non-ensemble algorithms were leveraged for prediction. The ensemble algorithms included Random Forest (RF), Bagging, and XG-Boost (added to the Weka software as an extension). The base algorithms also included Artificial Neural Network (ANN), Decision Tree (DT), and Support Vector Machine (SVM). These algorithms were selected based on their popularity in high-performing capability for prediction purposes and their extensive use in studies on healthcare topics [34–36].

To evaluate the performance efficiency, we utilized some performance criteria, including positive predictive value (PPV), negative predictive value (NPV), sensitivity, specificity, accuracy, F-Score, and Area Under the Receiver Operator Characteristics (AU-ROC) curve due to their numerous applications in most previous studies on biomedical research such as medicine [37–39]. We tested the current algorithms’ performance using various hyperparameter combinations with the Grid Search method to gain the best performance results. Also, we utilized one database containing 52 PC-confirmed cases from the Imam Khomeini Hospital of Sari City to test ML algorithms’ external validity and generalizability. We fed these data to the best model obtained for predicting the mortality risk of PC. Then, we measured the predictive strength of the selected model on these new cases as the external validation cohort.

### K fold cross-validation

One efficient resampling method in ML or data mining techniques for the proper prediction error of algorithms when tuning is using the K fold cross process [40]. As a data-splitting strategy, this method splits the data into K folds for training and testing the algorithms during the learning process. One fold is utilized for training the algorithms, and the rest of the data (K-1) is considered for training [41]. This process is performed in K times with random sampling with replacement [42]. The accuracy of the algorithms in this condition is equal to the average performance in all K times [43]. Also, due to the existing imbalance in the class numbers, it may be that not choosing the samples belonging to the minority class during the sampling technique; hence, a stratified type of K fold cross validation should be performed for selecting the samples according to the frequency of samples belonging to each class types [42, 44]. In the current study, we used stratified 10-fold cross-validation as a data-splitting strategy to establish the prediction models based on ML algorithms.

## Results

### Database preparation

**Fig. 1:**
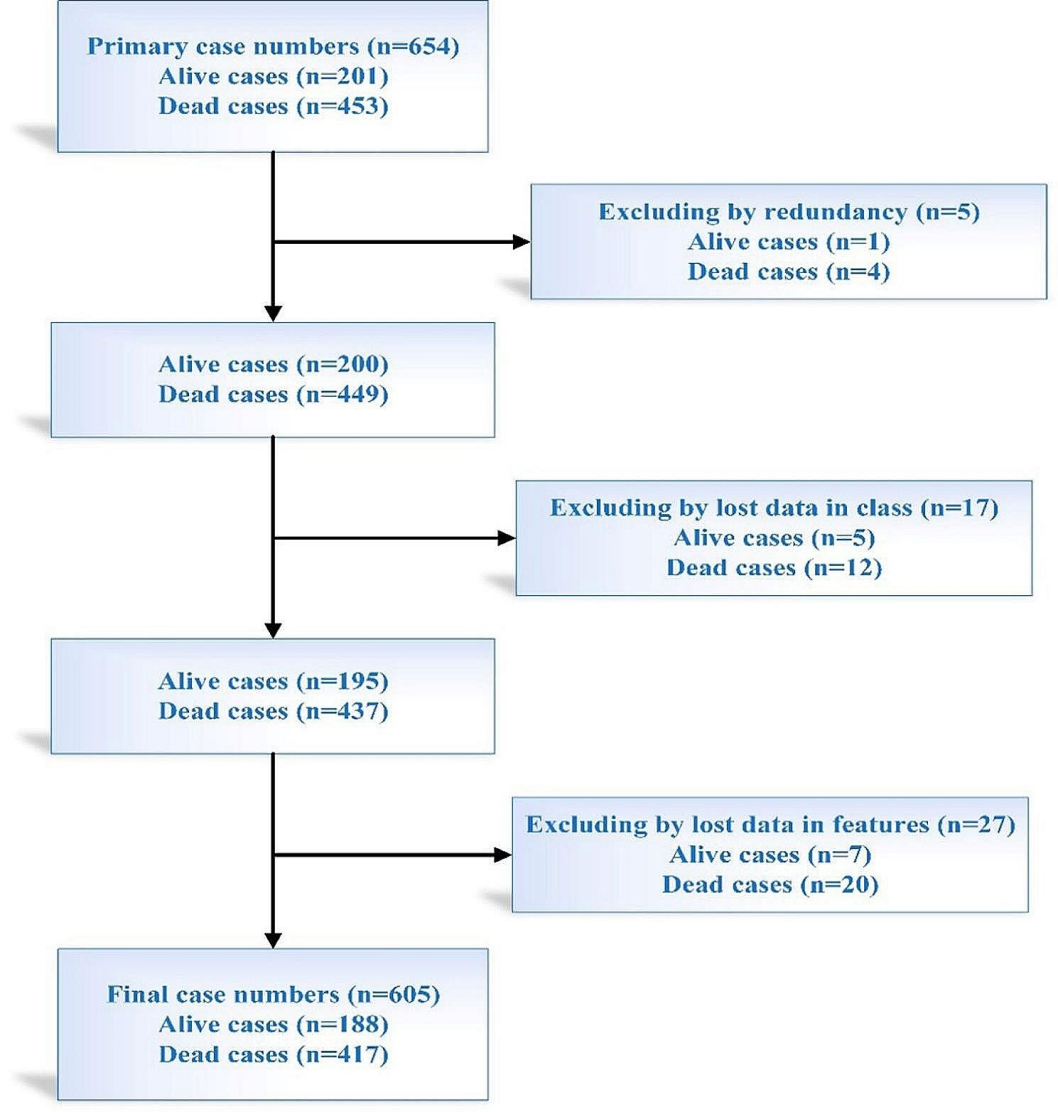
The preprocessing steps of the current database

After investigating the cases regarding redundancy, we removed five similar cases from the current database. By eliminating the records with missing data in their output class, 5 and 12 cases related to alive and dead cases were excluded from the present study. After examining the prognostic factors of the cases, 7 and 20 rows of alive and dead cases with more than 10% missing data were excluded from the current database. The lost data of the 15 and 35 cases related to alive and dead patients with less than 10% missing data were filled by the values of the same features in almost identical cases by the KNN algorithm. In this way of filling cases, the bias in ML models’ performance would be decreased compared to other lost data filling methods, such as using the average or mode of values. The flowchart of the excluding process and obtaining the final cases for data analysis in the current study is depicted in Fig. 1. Based on Fig. 1, the final cases for analysis in the current study included 605 cases divided into 188 and 417 ones belonging to alive and dead patients, respectively. The details of descriptive statistics of cases used for analysis are presented in Table 1.

**Table 1:** The characteristics of cases associated with alive and dead PC patients

Features	Values	Total cases (*n* = 605)	Alive cases (*n* = 188)	Dead cases (*n* = 417)	*P*-value
Age at diagnosis	< 55 55–65 > 65	116 214 275	48 85 55	68 129 220	0.01*
Gender	Male Female	316 289	95 93	221 196	0.1
Race	Persian Non-Persian	421 184	128 60	293 124	0.08
Residence status	Rural Urban	385 220	132 56	253 164	0.1
BMI	<=25 > 25	354 251	117 71	237 180	0.06
Smoking	Yes No	398 207	105 83	293 124	0.01*
Alcohol consumption	Yes No	93 512	45 143	48 369	0.01*
History of gastrointestinal cancer	Yes No	214 391	87 101	127 290	0.01*
History of other cancers	Yes No	252 353	96 92	156 261	0.01*
Surgery	Yes No	506 99	167 21	339 78	< 0.001*
Chemotherapy	Yes No	424 181	172 16	252 165	< 0.001*
Radiotherapy	Yes No	396 209	145 43	251 166	< 0.001*
Grade of tumor	Grade 1 (Well differentiated), Grade 2 (Moderately differentiated), Grade 3 (Poorly differentiated), Grade 4 (Undifferentiated)	88 247 213 57	12 57 89 30	76 190 124 27	< 0.001*
T-stage (Tumor size)	T1 (< 2 cm) T2 (2–4 cm) T3 (> 4 cm) T4 (grows outside the pancreas)	112 216 225 52	84 53 30 21	28 163 195 31	< 0.001*
N-stage (Lymph node invasion)	N0 (not spread to nearby lymph nodes), N1 (spread to no more than 3 nearby lymph nodes), N2 (spread to 4 or more nearby lymph nodes)	65 283 257	54 87 47	11 196 210	< 0.001*
M-stage (Metastasis state)	M0 (no distant sites spread), M1 (distant sites spread)	197 408	89 99	108 309	< 0.001*
Histological type	Adenocarcinoma, Squamous cell carcinoma, Other types	482 106 17	130 45 13	352 61 4	0.01*
Vascular invasion	Yes No	411 194	86 102	325 92	< 0.001*

As Table 1 shows, the prognostic factors including age at diagnosis, smoking, alcohol consumption, history of gastrointestinal cancer, history of other cancers, surgery, radiotherapy, chemotherapy, grade of tumor, tumor size, lymph node invasion, metastasis state, histological type, and vascular invasion revealed difference among alive and dead PC cases (*P* < 0.05). On the contrary, the factors, including gender, race, residence status, and BMI, didn’t differ statistically between the two groups.

### Feature selection

The results of scoring the prognostic factors for predicting the mortality risk of PC based on the binary logistic regression are shown in Table 2.

**Table 2:** The results of scoring prognostic factors for mortality risk of PC

Features	β*	OR**	95% CI*** of OR	*P*-value
Age at diagnosis	0.643	1.574	[1.226–2.119]	< 0.001*
Gender	0.126	1.077	[0.752–1.216]	0.689
Race	-0.238	0.871	[0.794–1.074]	0.293
Residence status	-0.197	0.894	[0.872–1.009]	0.384
BMI	-4.527	0.683	[0.547–0.824]	0.01*
Smoking	1.129	2.221	[1.876–3.455]	< 0.001*
Alcohol consumption	0.345	1.116	[0.942–1.198]	0.163
History of gastrointestinal cancer	0.548	1.135	[1.083–1.255]	< 0.001*
History of other cancers	0.433	1.052	[1.006–1.211]	< 0.001*
Surgery	0.876	1.524	[1.398–1.894]	< 0.001*
Chemotherapy	1.16	1.893	[1.348–2.585]	< 0.001*
Radiotherapy	0.733	1.389	[1.131–2.074]	< 0.001*
Grade of tumor	0.527	1.241	[1.152–1.375]	< 0.001*
T-stage (Tumor size)	0.473	1.197	[1.094–1.304]	< 0.001*
N-stage (Lymph node invasion)	0.455	1.149	[1.055–1.201]	< 0.001*
M-stage (Metastasis state)	0.672	1.316	[1.214–1.476]	< 0.001*
Histological type	0.395	1.159	[1.131–1.287]	< 0.001*
Vascular invasion	0.447	1.224	[1.076–1.443]	< 0.001*

*Regression coefficient, **Odd ratio, ***Confidence interval

Based on Table 2, the prognostic factors including, age at diagnosis (β = 0.643, OR = 1.574, 95% CI= [1.226–2.119]), BMI (β=-4.527, OR = 0.683, 95% CI= [0.547–0.824](, smoking(β = 1.129, OR = 2.221, 95% CI= [1.876–3.455]), history of gastrointestinal cancer(β = 0.548, OR = 1.135, 95% CI= [1.083–1.255](, history of other cancers(β = 0.433, OR = 1.052, 95% CI= [1.006–1.211](, surgery (β = 0.876, OR = 1.524, 95% CI= [1.398–1.894](, chemotherapy(β = 1.16, OR = 1.893, 95% CI= [1.348–2.585]), radiotherapy(β = 0.733, OR = 1.389, 95% CI= [1.131–2.074]), grade of tumor(β = 0.527, OR = 1.241, 95% CI= [1.152–1.375]), tumor size(β = 0.473, OR = 1.197, 95% CI= [1.094–1.304]), lymph node invasion(β = 0.455, OR = 1.149, 95% CI= [1.055–1.201]), metastasis state(β = 0.672, OR = 1.316, 95% CI= [1.214–1.476]), histological type(β = 0.395, OR = 1.159, 95% CI= [1.131–1.287]), and vascular invasion (β = 0.447, OR = 1.224, 95% CI= [1.076–1.443]) with correlation at *P* < 0.05 were considered as the essential factors for mortality risk of PC. On the contrary, gender, race, residence status, and alcohol consumption didn’t obtain competency statistically, so they were excluded from the further steps.

### Model development and evaluation

Table 3 shows the performance measurement results based on various performance criteria of chosen ML algorithms for predicting the mortality risk of PC. As mentioned, the performance results were reported based on 10-fold cross-validation in the best hyperparameters adjusted. Table 4 also shows the ranges of hyperparameters used to obtain the best-performing model for mortality risk prediction based on the Grid search technique.

**Table 3:** The performance results of the selected ML algorithms

Algorithm	Best hyperparameters tuned	PPV (%)	NPV (%)	Sensitivity (%)	Specificity (%)	Accuracy (%)	F-Score (%)
ANN	learning rate = 0.5, maximum epoch = 150, Hidden layers = 10	78.21	44.53	67.15	58.51	64.46	72.26
Bagging	Base classifier = Rep-Tree, number of iterations = 20, calculate out of bag = false	89.89	61.04	76.74	80.85	78.02	82.79
DT	Confidence factor = 0.25, binary splitting = false, minimum number of object = 1	80.00	47.92	70.02	61.17	67.27	74.68
RF	Maximum depth = 8, number of estimators = 10, maximum number of features = 6, maximum leaf nodes = 2	93.47	73.42	85.85	86.70	86.12	89.50
SVM	Kernel type = RBF, RBF gamma = 0.5, regression precision = 0.2, Control parameter (C) = 10	95.02	82.76	91.61	89.36	90.91	93.28
XG-Boost	Maximum depth = 10, eta = 0.1, Booster = gradient boosted tree	97.06	89.34	94.96	93.62	94.55	96.00

**Table 4:** The ranges of hyperparameters used as Grid search

Algorithm	Ranges of hyperparameters
ANN	Learning rate [0.3-1], maximum epoch [100–1000], number of hidden layers [6–30]
Bagging	Base classifier [J-48, Rep-tree, Random-tree], number of iterations [10–50], calculate out of bag [false, true]
DT	Confidence factor [0.15–0.3], binary splitting [false, true], minimum number of objects [1–3]
RF	Maximum depth [6–15], number of estimators [5–20], maximum number of features [5–10], maximum leaf nodes [1–4]
SVM	RBF gamma [0.3-1], Control parameter (C) [5–30], regression precision [0.1–0.5]
XG-Boost	Maximum depth [8–20], eta [0.1–0.5]

Based on Tables 3 and 4, the XG-Boost with PPVof 97.06%, NPV of 89.34%, sensitivity of 94.96%, specificity of 93.62%, accuracy of 94.55%, and F-Score of 96.00% with a maximum depth of 10 in tress, eta of 0.1, and gradient tree as booster was recognized as the best-performing model for predicting the mortality risk among PC patients. Also, the SVM (second rank) and RF (third rank) algorithms with performance results of more than 80% in all performance criteria obtained favorable performance in this respect. The bagging and DT algorithms obtained the fourth and fifth ranks for prediction purposes. The lowest performance was related to the ANN algorithm with PPVof 78.21%, NPV of 44.53%, sensitivity of 67.15%, specificity of 58.51%, accuracy of 64.46%, and F-Score of 72.26% for predicting the mortality risk.

**Fig. 2:**
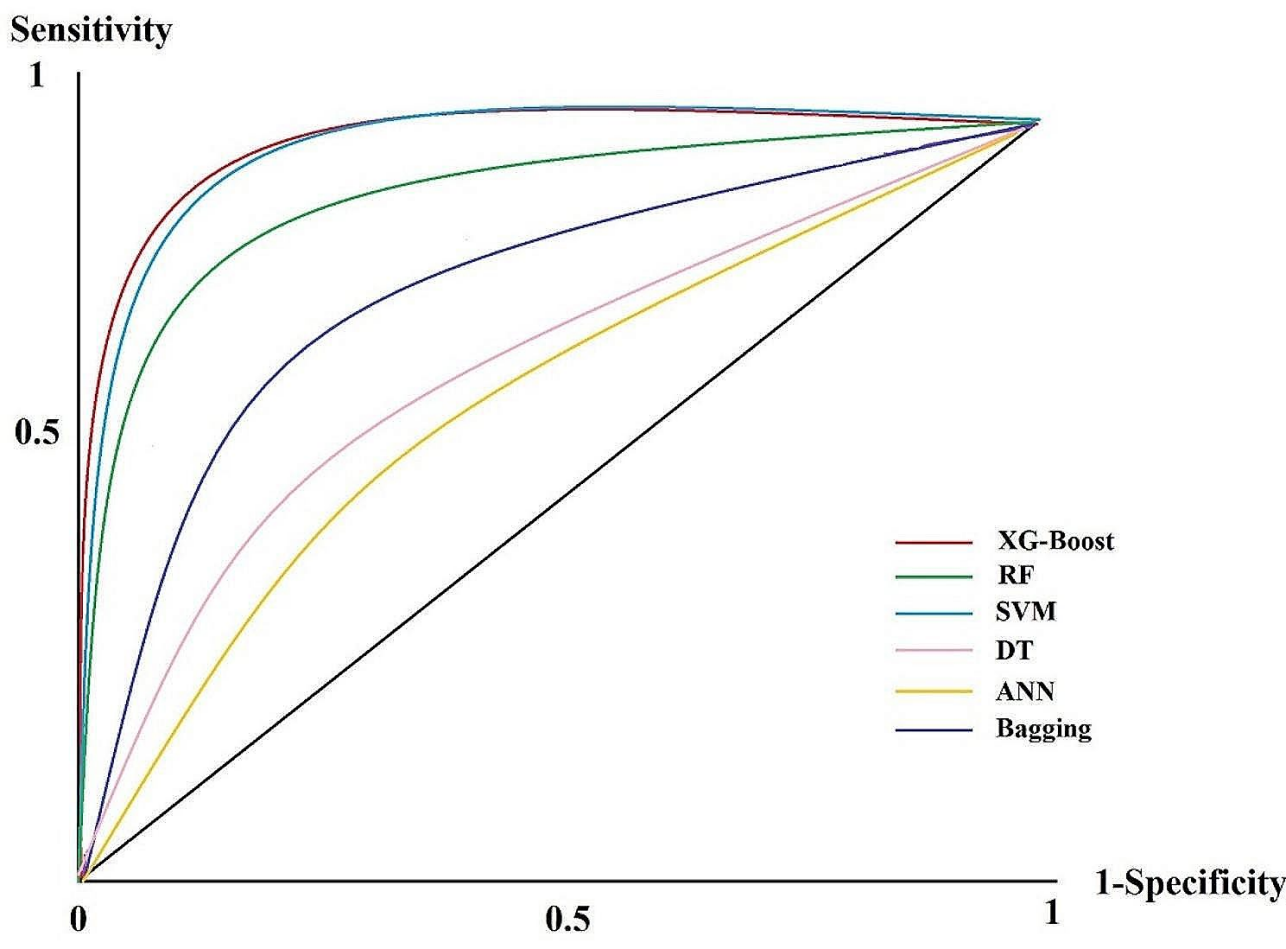
The ROC diagram of the ML-trained algorithms

Figure 2 shows the performance measurement of the chosen algorithms for predicting the mortality risk based on the AU-ROC curve. The random classifier line is placed between the sensitivity and 1-specificity vertices (black line).

Based on Fig. 2, the XG-Boost algorithm with AU-ROC of 0.933 (95% CI= [0.906–0.958]) revealed more competency than other ML-trained algorithms for predicting the mortality risk among PC patients (farther distance from the chance line in ROC curve). The next ML algorithm concerning performance strength was SVM with AU-ROC of 0.917 (95% CI [0.893–0.948]). The third, fourth, and fifth ranks in performance were associated with the RF, bagging, and DT, respectively. The worst performance from the ROC curve was obtained from the ANN algorithm with AU-ROC of 0.672 (95% CI =[0.663–0.705]) (the ROC curve was closer to the random classifier line than others).

### External validation cohort

**Fig. 3:**
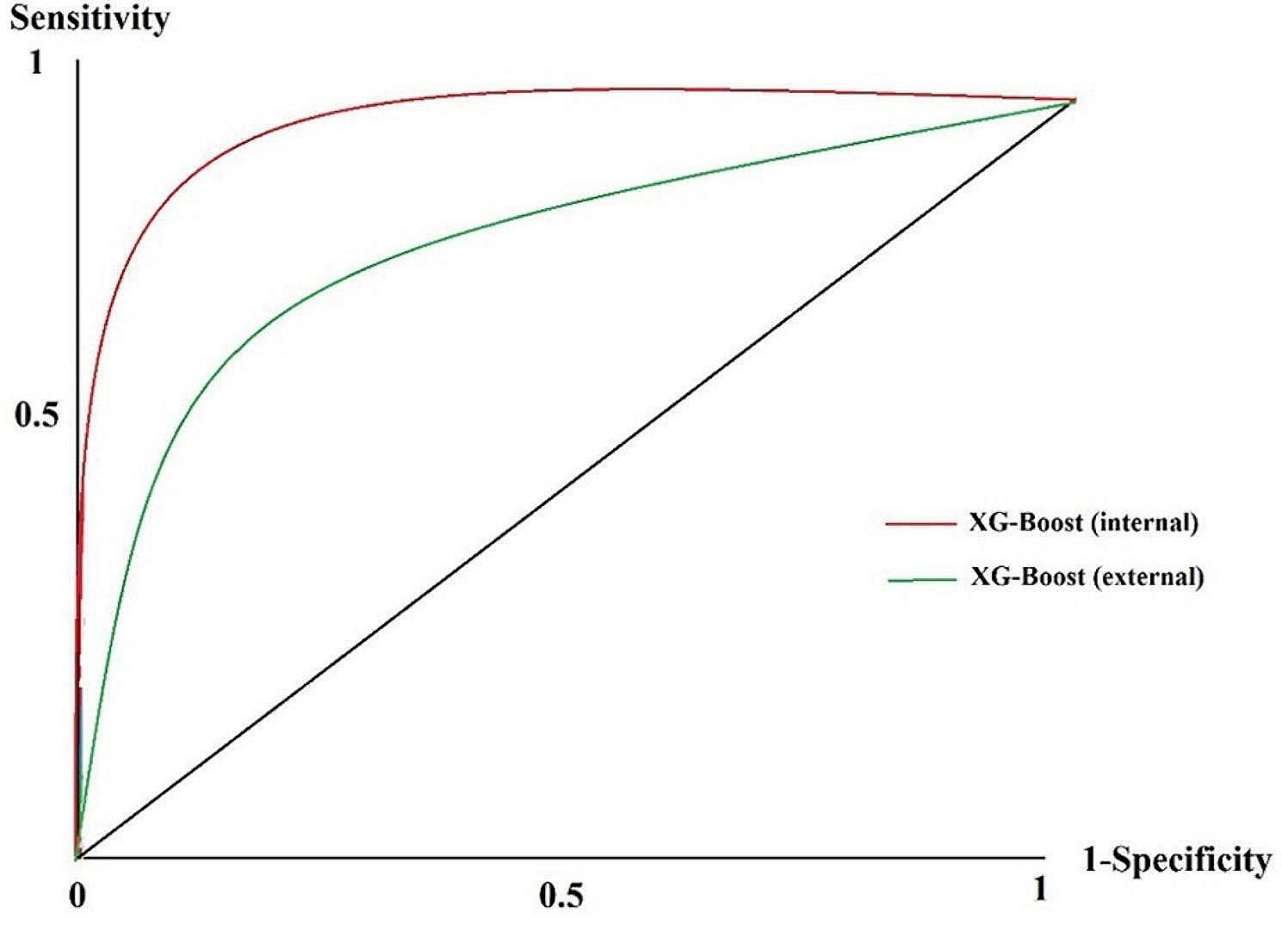
The ROC diagram of the XG-Boost algorithm in internal and external validation states

As mentioned in the methods section, we used 52 positive PC cases to test the generalizability and the strength of the current prediction model based on ML in other clinical centers. The 21 and 31 cases were associated with PC alive and dead cases, respectively. Classifying these cases using the XG-Boost as the best-performing model for prediction purposes revealed that this algorithm acquired TP = 26, FN = 5, FP = 6, and TN = 15. We used the ROC curve to compare two internal and external validation modes regarding performance efficiency. The results of the ROC curve in two modes of XG-Boost are depicted in Fig. 3.

According to Fig. 3, the XG-Boost in external validation condition obtained an AU-ROC of 0.836 (95% CI= [0.789–0.865]. The results of the external validation of this algorithm showed an average performance reduction of 0.1 compared to the internal state (AU-ROC = 0.933 (95% CI= [0.906–0.958])), indicating the favorable performance in external validity. Therefore, the XG-Boost demonstrated desirable generalizability based on these external cases.

### Variable importance

**Fig. 4:**
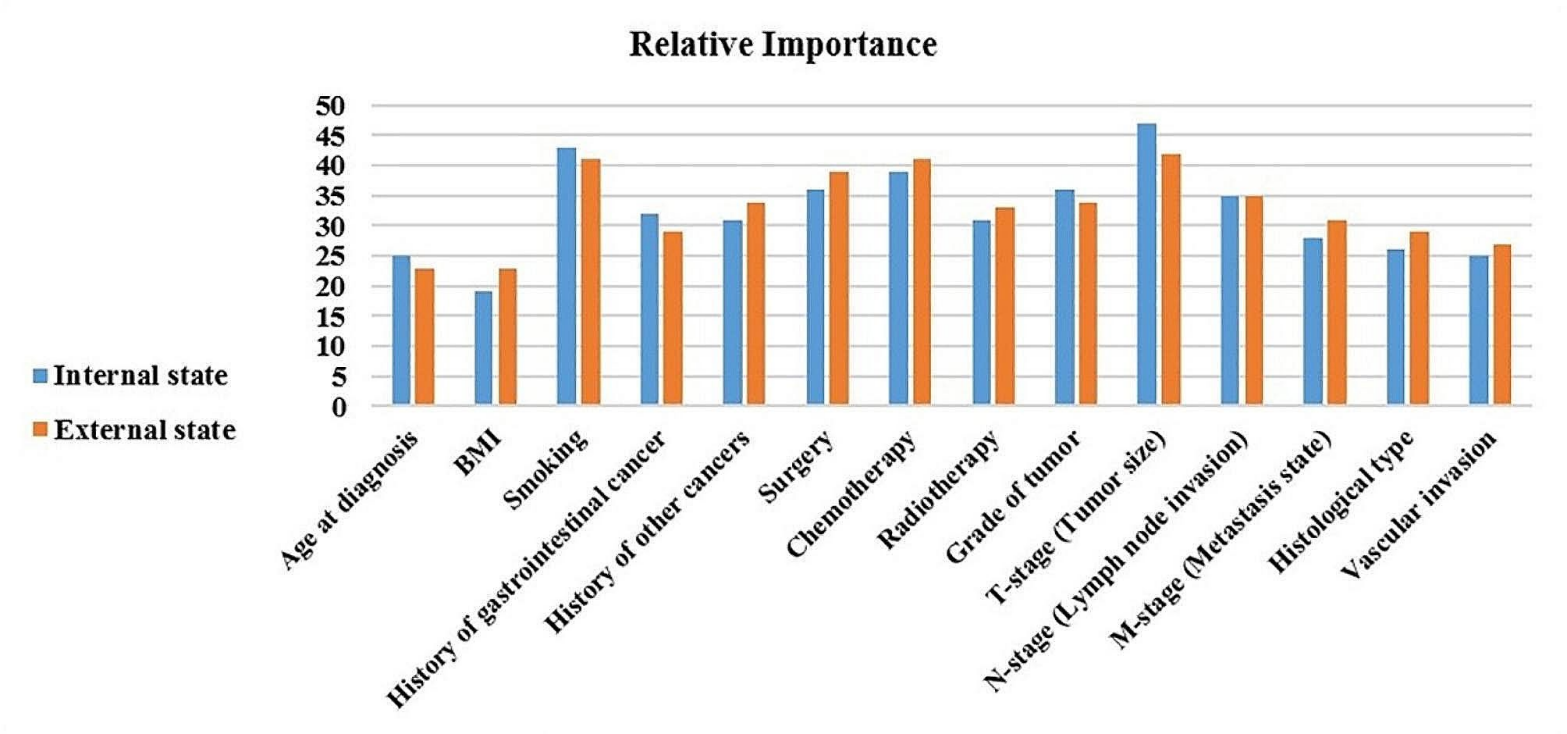
The relative importance of factors influencing the mortality risk

In the current study, we utilized the XG-Boost model to score and assess the impact of the prognostic factors on the prediction of the mortality risk of PC in internal and external modes. The relative impact is considered the commonly used method in ML techniques to assess the importance of each feature on the outcome variable [45]. The results of scoring the prognostic factors based on the relative importance (RI) score gained by XG-Boost in two modes are illustrated in Fig. 4.

As Fig. 4 shows, the factors including tumor size with RI of 47 and 45 in internal and external modes, smoking with RI of 43 and 41 in internal and external modes, and chemotherapy with RI of 39 and 41 in internal and external states were regarded as the best prognostic factors for mortality risk of PC among patients. On the contrary, the factors, including age at diagnosis with RI of 25 and 23 in internal and external states and BMI with RI of 19 and 23 in internal and external states, obtained less impact on the prediction purposes.

## Discussion

The current study aims to build a prediction model to predict the mortality risk of PC among patients by using ML approaches and prognostic factors. To achieve this, we applied one single-centered database containing prognostic factors. We first leveraged the feature selection technique with the help of binary logistic regression to obtain crucial prognostic factors. Then, we used the chosen ensemble and non-ensemble ML algorithms, including ANN, bagging, DT, RF, SVM, and XG-Boost, to establish the prediction models for the mortality risk of PC among patients. Also, we applied the external clinical data to test the generalizability strength of the current prediction model in other clinical environments. Finally, we assessed the prognostic factors using the XG-Boost algorithm, known as the best-performing model in the current study, in internal and external datasets. The results of the current study demonstrated that the XG-Boost with PPV of 97.06%, NPV of 89.34%, sensitivity of 94.96%, specificity of 93.62%, accuracy of 94.55%, F-Score of 96%, and AU-ROC of 0.933 (95% CI= [0.906–0.958]) was considered as the best-performing model for predicting the mortality risk. Also, this algorithm with AU-ROC of 0.836 (95% CI= [0.789–0.865] gave us an almost favorable performance in the external validation state. Based on the XG-Boost, the prognostic factors including tumor size with RI of 47 and 45 in internal and external modes, smoking with RI of 43 and 41 in internal and external modes, and chemotherapy with RI of 39 and 41 in internal and external states were found as the best prognostic factors for predicting the mortality risk of PC among patients.

So far, few studies have been conducted on leveraging ML techniques to predict the mortality risk of PC. Sun et al. conducted ML research to predict the specific mortality of PC. They used SEER data, including the prognostic factors. The algorithms in their study consisted of Cox hazards, random and conditional inference survival forests, and DeepHit. Their study demonstrated that the survival quilt model with a C-index of 0.726 and the Cox model with a C-index of 0.698 and 0.695 obtained the best performance for predicting the 1-year, 3-year, and 5-year mortality among PC patients, respectively [46]. In the current study, the XG-Boost as an ensemble algorithm with AU-ROC of 0.933 (95% CI= [0.906–0.958]) and AU-ROC of 0.836 (95% CI= [0.789–0.865] obtained the favorable performance in internal and external modes, respectively. Also, lifestyle factors such as smoking with RI of 43 and 41 in internal and external modes represented satisfactory predictability in the current study that wasn’t considered in the Sun’s study.

Also, some studies have been conducted on the survival assessment of PC based on ML techniques. Baek et al. utilized ML approaches to predict PC survival using multi-omics data. They showed that the logistic regression with AU-ROC of 0.769 obtained better performance than other ML algorithms [47]. In the current study, the XG-Boost as an ensemble ML technique with AU-ROC of 0.933 (95% CI= [0.906–0.958]) seems that obtained a better performance than Baek’s study.

Keyl et al. presented ML algorithms as a solution to better predict the survival of the advanced PC. The survival rate of PC was 6.7 months with a confidence interval of 5.8-0.86 months. In their study, the random survival forest with a C-index of 0.71 achieved more competency than other ML approaches by using clinical data of 203 PC patients [48]. In their study, they applied some laboratory and molecular characteristics, such as C-reactive protein and neutrophil-to-lymphocyte ratio, that didn’t exist in our database for analysis. Also, they ignored some lifestyle factors, including smoking and alcohol consumption. Walczak et al. leveraged the ANN to predict the survival of PC based on the data from 219 patient records. According to their results, the ANN obtained the sensitivity and specificity of 91% and 38%, respectively, for the prediction of 7-month survival of PC [49]. In the current study, the ANN, with a sensitivity and specificity of 67.15% and 58.51%, obtained the lowest performance for the prediction purpose. On the contrary, the XG-Boost, with a sensitivity of 94.96% and specificity of 93.62%, gained more prognostic competency than Walczak’s study. In summary, ML techniques played a significant role in other modes associated with PC disease, such as predicting the risk determination and early detection of PC [50–53], quality of life and surgical outcomes after surgery [54], death after surgery [55], risk of recurrence [56], and differentiating the tumor types [57].

One strength of the current study that has not been addressed in most previous studies is using a native database to develop a model to predict PC mortality risk. Moreover, the external validation of the trained algorithm revealed almost desirable performance in another clinical environment, implying the clinical applicability of the current prediction model in another clinical environment in our country. Another strength of the current study was leveraging the database, including some lifestyle factors, such as smoking, which were recognized as having a significant role in the prediction purpose and were not considered in other studies. Using the external validation cohort is one common way to estimate the bias and generalizability for prediction purposes, and it was considered in the current research. The clinical applicability of the established prediction model can be regarded as utilizing the best-performing prediction model as a knowledge base of intelligent systems to estimate the mortality risk of PC patients based on prognostic factors by doctors. Therefore, the high-risk PC group would be evaluated by various prognostic factors. In the following steps, clinical solutions such as multiple treatments, screening, and prevention measures can be performed to mitigate the mortality risk of PC by enhancing the patient status regarding the prognostic factors, especially the modifiable ones contributing to the high mortality risk.

### Limitations and future directions

While performing the current study, we confronted some limitations that should be addressed. 1-Some data were filled through an imputation process that may affect the generalizability of the prediction models to some extent; hence, we suggest filling the lost values with actual data from the records as much as possible. 2- The database used for the current study was retrospective and single-centered. We recommend the cohort and multi-centered database to establish the prediction model for better accuracy and generalizability. 3- Some factors, including laboratory and omics data, were used for prediction in other studies. We recommend using these factors to gain more accuracy and interoperability of ML models in other clinical environments. 4- For the external validation test, we applied a small sample of data from PC patients due to the impossibility of collecting more data, limiting a full judgment on the generalizability of the current prediction model.

## Conclusion

In the current study, we utilized ML techniques to build the prediction model for predicting the mortality risk of PC. We concluded that the XG-Boost with AU-ROC of 0.933 (95% CI= [0.906–0.958]) and 0.836 (95% CI= [0.789–0.865] gained a favorable performance for the prediction using the internal and external data. Based on the XG-Boost, the factors including tumor size, smoking, and chemotherapy were considered the top factors for predicting the mortality risk of PC among patients. Superior features can help clinicians understand predictive outcomes and support them as decision-makers in achieving personalized decisions more efficiently. Although the computational logic in XG-Boost is vague in predicting outcomes from features, this algorithm can be used as an efficient knowledge base for intelligent systems to be used by clinicians in clinical environments to assess patients’ clinical modes. Obtaining essential factors by XG-Boost and its application in intelligent systems can play a significant role for doctors to focus more on these factors after evaluating patients’ risk and introduce more appropriate individual decisions for a better prognosis. In this way, for the patients categorized as high-risk groups, the best preventive, diagnostic, or therapy measures can be achieved based on these factors, especially the modifiable ones.

## Data Availability

The datasets used and/or analysed during the current study available from the corresponding author on reasonable request.
